# Amide Proton Transfer-weighted MRI in the Diagnosis of Major Salivary Gland Tumors

**DOI:** 10.1038/s41598-019-44820-0

**Published:** 2019-06-06

**Authors:** Yun Jung Bae, Byung Se Choi, Woo-Jin Jeong, Young Ho Jung, Jung Hyun Park, Leonard Sunwoo, Cheolkyu Jung, Jae Hyoung Kim

**Affiliations:** 1 Department of Radiology, Seoul National University Bundang Hospital, 82, Gumi-ro 173beon-gil Bundang-gu, Seongnam, Republic of Korea; 2 Department of Otolaryngology–Head & Neck Surgery, Seoul National University Bundang Hospital, 82, Gumi-ro 173beon-gil Bundang-gu, Seongnam, Republic of Korea

**Keywords:** Diagnostic markers, Tumour biomarkers, Cancer imaging

## Abstract

Amide proton transfer-weighted magnetic resonance imaging (APTw-MRI), which is effective in tumor characterization, has expanded its role in the head and neck. We aimed to evaluate the diagnostic ability of APTw-MRI in differentiating malignant from benign major salivary gland tumors compared with diffusion-weighted imaging (DWI) and dynamic contrast-enhanced (DCE)-MRI. Between December 2017 and November 2018, 38 subjects, who were diagnosed with major salivary gland tumors and who underwent preoperative 3 T MRI, including APTw-MRI, DWI, and DCE-MRI, were included in this retrospective study. Twenty-three subjects had benign tumors, and fifteen had malignancies. APTw-signals of the tumors were measured and compared according to the histopathological diagnosis. Using receiver operating characteristic curve analysis, diagnostic performance of APTw-MRI was evaluated and compared with DWI and DCE-MRI using DeLong test. The maximum, mean, and median APTw-signals were significantly higher in malignant than in benign tumors (*P* < 0.001). The mean and maximum APTw-signals showed excellent area under the curve for predicting malignant tumors (0.948 and 0.939), which were significantly higher than the combining use of DWI and DCE-MRI (0.780) (*P* = 0.021 and 0.028). Therefore, APTw-MRI could be a useful tool for differentiating malignant from benign major salivary gland tumors, and can be applicable in the clinical setting.

## Introduction

Salivary gland tumors account for approximately 2–5% of all tumors of the head and neck^1,2^. They mostly arise in the major salivary glands, and nearly 80% of them in the parotid glands^2^. Strategy for treatment can vary depending on tumor characteristics; aggressive surgical procedure is required for malignant tumors, whereas some benign tumors can be followed-up without surgical intervention when properly diagnosed^3,4^. Therefore, accurate discrimination between benign and malignant tumors in preoperative setting is important in treatment planning for tumors in the major salivary glands^3^.

Magnetic resonance imaging (MRI) is superb in assessing head and neck tumors with good visualization of the soft-tissue and high spatial resolution^2,5^. However, conventional MRI, such as T1-weighted imaging (T1-WI) and T2-WI, has been known to have relatively low diagnostic accuracy^3,6^. Rather, multi-parametric analysis using functional MRI parameters, including apparent diffusion coefficient (ADC) from diffusion-weighted imaging (DWI) and time-intensity curve (TIC) from dynamic contrast-enhanced (DCE)-MRI, has been proven to be effective in the differentiation with high diagnostic accuracy^3,7^. However, its clinical application can be burdensome, because many image acquisitions, followed by exogenous contrast agent injection, post-processing, and multiple stepwise decisions, are required.

Recently, amide proton transfer-weighted (APTw)-MRI has been introduced as a new endogenous contrast mechanism for molecular imaging. It can detect amide proton constituents abundant in tumors, based on the effect of chemical exchange saturation transfer between free water and mobile proteins/peptides backbones^5,8–10^. Using this property, APTw-MRI can detect and characterize various tumors of the brain^10–15^, prostate^16^, breast^17^, lung^18^, rectum^19^, and endometrium^20^. However, regarding the head and neck region, the use of APTw-MRI has been limited due to susceptibility artifacts from air-containing structures and motions^5^. Although a few studies showed that APTw-MRI could be feasible for the use in the head and neck^5,21^, there is no study evaluating its feasibility for salivary gland tumors to date.

In this study, we adopted APTw-MRI in the preoperative assessment of parotid and submandibular gland tumors. We believed that parotid and submandibular spaces could be eligible for APTw-MRI with tolerable field inhomogeneity. We sought to assess the diagnostic performance of APTw-MRI in differentiating malignant from benign salivary tumors, and compared it with multi-parametric analysis using DWI and/or DCE-MRI. The purpose of this study was to determine the utility of APTw-MRI in differentiating between benign and malignant major salivary gland tumors compared with DWI and/or DCE-MRI.

## Results

### Demographics

There was no significant difference in age of the patients between benign (median 56 years, range 18–72 years) and malignant (62 years, 38–78 years) tumors (*P* = 0.055). Also, sex of the patients did not differ significantly between benign (15 females and 8 males) and malignant (5 females and 10 males) tumors (*P* = 0.096).

### Inter-observer agreement of APTw-signal measurements

The inter-observer agreements between the two readers were excellent for maximum (intraclass correlation coefficient [ICC], 0.995; 95% confidence interval [CI], 0.991–0.998), mean (0.841; 0.679–0.922), median (0.796; 0.588–0.899), and minimum (0.767; 0.527–0.885) APTw-signal values. The inter-observer agreement was good for kurtosis (0.614; 0.218–0.809) and skewness (0.602; 0.195–0.804).

### APTw-MRI findings in benign and malignant tumors

**Figure 1:**
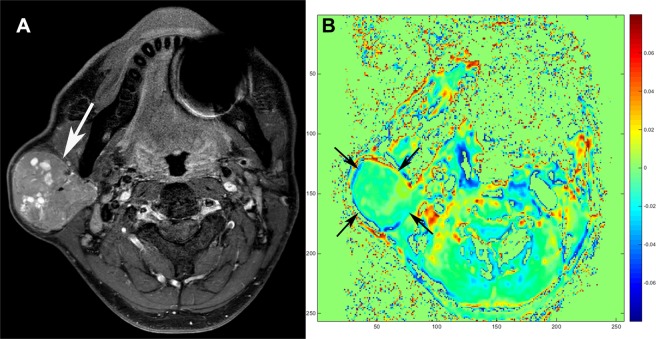
Warthin tumor of a 46-year-old male. (**A**) Axial T2-weighted imaging with fat suppression shows a well-defined hyperintense mass with multifocal cystic portion in the right parotid gland (arrow). (**B**) Amide proton transfer-weighted (APTw)-MRI demonstrates that APTw-signal of the tumor is relatively low (arrows). The averaged APTw-signal values obtained by the two readers in the solid portion were as follows: Mean APTw-signal, −2.36%; maximum APTw-signal, −0.53%; median APTw-signal, −2.35%.

**Figure 2:**
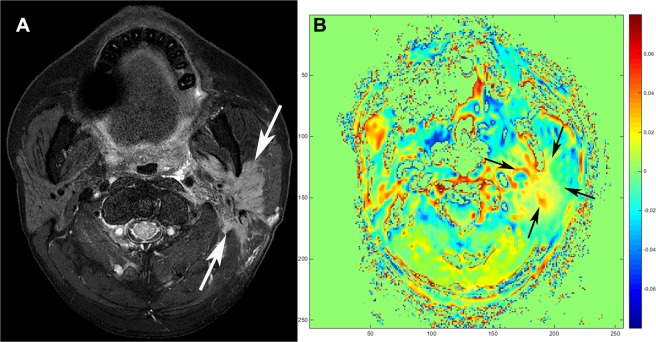
Squamous cell carcinoma of a 55-year-old male. (**A**) Axial T2-weighted imaging with fat suppression shows an irregular mass with infiltrative margin and heterogeneous signal intensities in the left parotid gland (arrows). (**B**) Tumor shows heterogeneous signal on amide proton transfer-weighted (APTw)-MRI (arrows). Note the markedly increased asymmetry value at the solid portion of the tumor. The averaged APTw-signal values from the two readers were as follows: Mean APTw-signal, 2.44%; maximum APTw-signal, 10.22%; median APTw-signal, −2.53%.

Table 1 and Supplementary Fig. 1 summarize the results of APTw-signal measurements. The mean, maximum, and median APTw-signals were significantly higher in malignant tumors than in benign tumors (all, *P* < 0.001) (Figs 1 and 2). Skewness and kurtosis were also significantly higher in malignant tumors than in benign tumors (*P* < 0.001 and *P* = 0.038). Minimum APTw-signal did not show significant difference between benign and malignant tumors (*P* = 0.224).

**Table 1:** APTw-signal values according to benign and malignant tumors

	Benign tumors	Malignant tumors	*P* values
Mean (%)	1.27 (−2.36, 4.22)	4.85 (0.059, 14.67)	<0.001*
Minimum (%)	−1.88 (−6.51, 0.96)	−0.52 (−9.67, 8.10)	0.224
Maximum (%)	4.00 (−0.53, 13.05)	10.27 (3.48, 40.15)	<0.001*
Median (%)	1.37 (−2.35, 4.42)	3.39 (0.062, 26.94)	<0.001*
Skewness	−0.010 (−0.72, 2.37)	0.78 (0.17, 2.05)	<0.001*
Kurtosis	0.18 (−1.21, 7.82)	0.88 (−0.24, 7.30)	0.038*

Note, Data are present as median (range).

APTw-signal = amide proton transfer-weighted signal.

^*^P values less than 0.05.

### DWI and/or DCE-MRI findings in benign and malignant tumors

On DWI, the mean ADC value was significantly lower in malignant tumors (median, 1.06 × 10^−3^ mm^2^/sec; range, 0.77 × 10^−3^–1.44 × 10^−3^ mm^2^/sec) than in benign tumors (1.62 × 10^−3^ mm^2^/sec; 0.61 × 10^−3^–2.77 × 10^−3^ mm^2^/sec) (*P* = 0.009). The TIC patterns from DCE-MRI was significantly different between benign (type A, n = 11; type B, n = 3; type C, n = 9; type D, n = 0) and malignant (type A, n = 3; type B, n = 0; type C, n = 12; type D, n = 0) tumors (*P* = 0.028). When categorizing tumors on the basis of multi-parametric analysis of DWI and DCE-MRI combining the use of ADC and TIC, 19 out of 23 benign tumors and 11 out of 15 malignant tumors were correctly determined.

### Comparison of diagnostic performance between APTw-MRI and DWI and/or DCE-MRI for malignant tumors

Regarding APTw-MRI, the receiver operating characteristic (ROC) curve analysis revealed that the diagnostic performance was excellent when using the mean (area under the curve [AUC], 0.948; 95% CI, 0.823–0.994) and maximum (0.939; 0.811–0.991) APTw-signals, followed by the median APTw-signal (0.916; 0.779–0.981), skewness (0.843; 0.689–0.941), and kurtosis (0.701; 0.531–0.839) (all, *P* < 0.001, except for kurtosis with *P* = 0.018).

As for DWI and/or DCE-MRI, the AUC was highest when adopting multi-parametric analysis combining ADC and TIC (0.780; 0.616–0.898; *P* < 0.001), followed by the ADC value alone (0.751; 0.584–0.876; *P* = 0.003), and TIC pattern alone (0.691, 0.521–0.831; *P* = 0.015).

**Figure 3:**
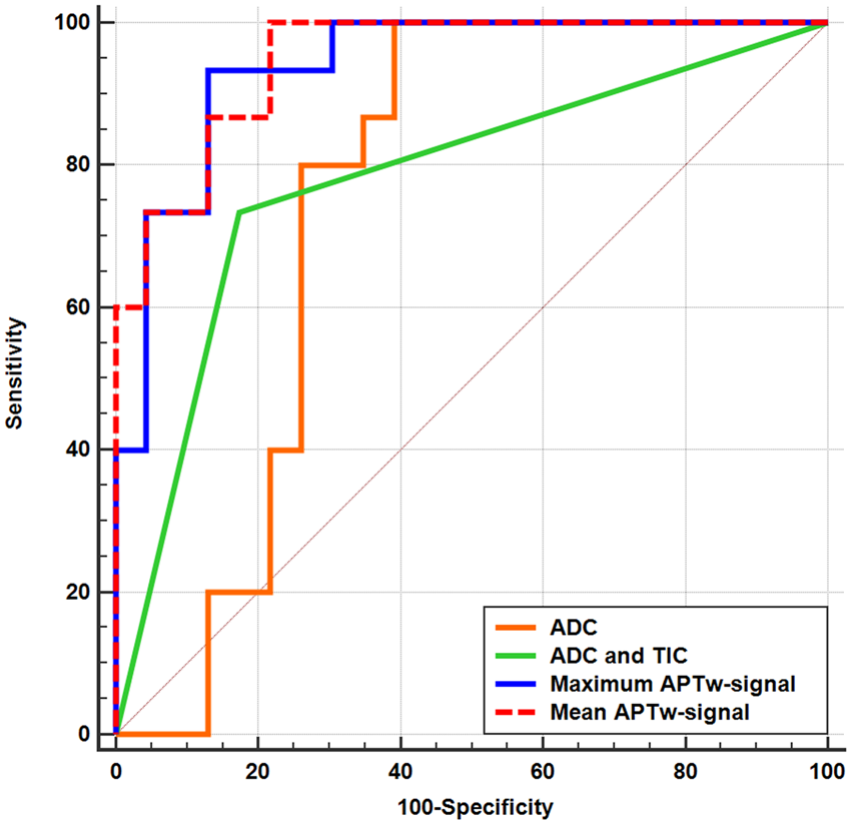
Receiver operating characteristic (ROC) curves of amide proton transfer-weighted (APTw)-MRI, diffusion-weighted imaging (DWI) and dynamic contrast-enhanced (DCE)-MRI in diagnosing malignant salivary gland tumors. ROC curves using mean APTw-signal (red dotted line, area under curve [AUC] 0.948), maximum APTw-signal (blue line, AUC 0.939), multi-parametric analysis of DWI and DCE-MRI combining apparent diffusion coefficient (ADC) and time-intensity curve (TIC) (green line, AUC 0.780), and ADC on DWI alone (orange line, AUC 0.751) are shown. Diagonal line represents AUC of 0.50. AUCs of mean APTw-signal and maximum APTw-signal are significantly higher than AUCs of DWI and/or DCE-MRI. (*P* = 0.021, 0.028, 0.02, and 0.05, respectively).

When we compared ROC curves from APTw-MRI and DWI and/or DCE-MRI using DeLong test, the AUCs for predicting malignancy were significantly higher with the use of mean and maximum APTw-signals than with the use of multi-parametric analysis (*P* = 0.021 and 0.028, respectively) and ADC alone (*P* = 0.02 and 0.05, respectively) (Fig. 3). The estimated sensitivity and specificity at the optimal cut-off level of each value are summarized in Table 2.

**Table 2:** Diagnostic performance of APTw-MRI and DWI and/or DCE-MRI for malignant tumors.

	Cut-off value	Sensitivity (%)	Specificity (%)
Mean APTw-signal	1.90%	100	78.3
Maximum APTw-signal	7.19%	93.3	87.0
Median APTw-signal	2.42%	80.0	91.3
Skewness	0.365	93.3	78.3
Kurtosis	0.270	86.7	56.5
ADC alone	1.44 × 10^−3^ mm^2^/sec	100	60.9
TIC alone	Type C	80.0	60.9
ADC and TIC	*Ref*.*	73.3	82.6

Note, APTw-MRI = amide proton transfer-weighted MRI, APTw-signal = amide proton transfer-weighted signal, DCE-MRI = dynamic contrast-enhanced magnetic resonance imaging, TIC = time-intensity curve.

*Ref, Refer to the subsection of “*DWI and DCE-MRI analysis*” of the “Materials and Methods” section.

## Discussion

In this current study, we revealed that the APTw-signals were significantly higher in malignant tumors arising in the major salivary glands than in benign tumors. Using the mean and maximum APTw-signals, excellent diagnostic performance in the prediction of malignant tumors was achieved, which was significantly higher than the multi-parametric analysis using DWI and/or DCE-MRI.

Preoperative diagnosis of major salivary gland tumors is important in surgical planning^3,4^. Fine needle aspiration – a common diagnostic procedure – is sometimes inconclusive due to sampling error or inaccessible tumor location^3,22,23^. Thus, the preoperative imaging becomes crucial in the diagnosis of major salivary gland tumors. However, conventional MRI features, such as signal intensity or tumor margin, have resulted in poor diagnostic outcomes^3,6^. A use of single functional parameter, such as ADC from DWI or TIC from DCE-MRI, can help precise diagnosis^24,25^. However, breakthrough was made by multi-parametric analysis with simultaneous usage of ADC and TIC, which showed high diagnostic accuracy in differentiating parotid tumors^3,7^.

Under these circumstances, we endeavored to find a simpler, yet more accurate, diagnostic method for major salivary gland tumors with APTw-MRI. APTw-MRI is an advanced imaging technique that can generate APTw-signal in proportion to tumor cellular density and/or proliferation without using exogenous contrast materials^8–10,18,20,26^. Relevantly, many studies have utilized APTw-MRI in cancer assessment. Currently, it is generally accepted that APTw-signal increases in malignancy than in benign tumor, and can be positively correlated with histologic grade^10–13,15–17,19,20,26,27^.

Nevertheless, the role of APTw-MRI in characterizing head and neck tumors has not been actively evaluated thus far. Until now, only a single study group published two preliminary results regarding the utility of APTw-MRI in the head and neck region^5,21^. In their most recent study^21^, the authors analyzed the APTw-signals in head and neck cancers, including nasopharyngeal undifferentiated carcinoma, squamous cell carcinoma, and lymphoma, and concluded that the mean and median APTw-signals of these malignancies were significantly higher than those of benign salivary gland tumors and normal tissues. This study well demonstrated that APTw-MRI could effectively identify head and neck cancers. However, the authors did not include any malignant tumors from the salivary gland, and they only compared 14 cases of benign salivary gland tumors with a heterogeneous group of head and neck malignancies, excluding those with salivary gland origin. Thus, the capability of APTw-MRI in differentiating malignant from benign salivary gland tumors remains to be elucidated.

In our study, we focused on the major salivary gland tumors, and found that APTw-signal values were significantly higher in malignant tumors in parotid and submandibular glands than in benign tumors. This result was in accordance with the previous studies that suggested APTw-signals in malignancies can increase higher than in benign tumors due to increased glandular tumor cells containing abundant mobile proteins/peptides, leading to an increased effect of chemical exchange saturation transfer^13,16,20,21,26^. In addition, skewness and kurtosis were significantly higher in malignant tumors than in benign tumors. This may reflect the underlying pathologic architecture of the malignant tumors, with a large number of pixels possessing high APTw-signals from malignant tissues. Moreover, APTw-MRI achieved excellent diagnostic performance for malignant tumors, even higher than multi-parametric analysis combining DWI and DCE-MRI (Supplementary Figs 2 and 3). It is worth noting that APTw-MRI has additional merit compared with DCE-MRI in that it can avoid exogenous contrast agent. APTw-MRI also can reduce the steps of image acquisition and decision-making processes compared with multi-parametric analysis combining DWI and DCE-MRI. Therefore, we could suggest that APTw-MRI may further add value in the assessment of major salivary gland tumors with clinical usefulness.

Compared with the previous studies that used DWI and DCE-MRI^3,7^, the overall sensitivity and specificity for the malignant tumors were lower in our study (sensitivity, 86% versus 73.3%; specificity, 92–100% versus 82.6%). We presume that this is likely due to our small study population, especially small number of malignant tumors. Another reason might be the differences in the image protocol for DWI and DCE-MRI. Thus, further validation of the optimal protocol for DWI and DCE-MRI as well as APTw-MRI is warranted.

There were several limitations in our study. First, the absolute magnitude of the percentage change in the APTw-signal depends on the imaging parameters of the pulse sequence^28^. Thereby, the absolute numbers of the APTw-signal from our study may differ from those from the previous studies^5,21^. However, when using the same imaging method, the MTR_asym_ (3.5 ppm) should always be higher in malignant tumors than in benign tumors^28^. Therefore, despite the differences in the absolute values, APTw-signal can be utilized in the differentiation of the malignant salivary gland tumors in the clinical setting. Second, as aforementioned, the number of our study population was small, and the sample size of malignant tumors was small compared with that of benign tumors; also, the malignant tumors consisted of various histological subtypes. Resultantly, the variation of APTw-signal was high in the malignant tumor group. However, since salivary gland tumors – especially malignant tumors – are not common, the low disease prevalence is the fundamental limiting factor. Furthermore, despite the heterogeneous histological types, our results showed that APTw-signals of the malignant tumors were significantly higher than those of the benign tumors. Since the decision of malignant versus benign tumor is more important than the histopathological diagnosis in the preoperative imaging, our result is relevant to the clinical practice. We believe that future study with more homogeneous and larger number of malignant tumors will reveal the improvement in the diagnostic performance with less signal variation. Third, we could not statistically analyze the added value of APTw-signals on DWI and DCE-MRI, also due to the small sample size. Therefore, further study with larger number of cases – especially malignant cases – is necessary to verify our results. Fourth, we did not exclude small-sized tumors from the analysis. Previous studies using APTw-MRI^5,21^ have excluded small-sized tumors to allow reliable signal measurements. However, when we measured the region-of-interest (ROI) areas of tumors, the ROIs ranged in size from 100.61 to 2034.89 mm^2^. We believe that this value was sufficient for the measurement of APTw-signals under in-plane resolution of 2 × 2.5 mm^2^. Therefore, this should have minimal effect on our study results. Fifth, the ROI allocation could be subjective depending on two different readers. However, we aimed to minimize the possible inter-reader variance in placing ROI with several strategies detailed in the methods. In addition, the inter-observer agreements of APTw-signal measurements between the two readers were confirmed to be excellent and/or good by ICC analysis. We believe that this result can reflect that the issue of subjective ROI drawing could be solved via systemized allocation.

In conclusion, APTw-signals were significantly higher in malignant tumors of the major salivary gland than in benign tumors. The diagnostic performance of APTw-MRI in predicting malignancy was excellent and superior to DWI and DCE-MRI. In addition, APTw-MRI is a technique that can generate APTw-signal without using exogenous contrast materials. Therefore, APTw-MRI could be a useful tool for discriminating malignant from benign tumors of the major salivary glands, and can be applicable in the clinical setting.

## Methods

### Subjects

This retrospective study was approved by the institutional review board of our institution, and written informed consent was waived. Between December 2017 and November 2018, 164 subjects underwent head and neck MRI for the evaluation of clinically suspected major salivary gland tumors. The inclusion criteria were as follows: *(a)* initial diagnosis of parotid or submandibular gland tumors, *(b)* pathologically proven tumors by fine needle aspiration, biopsy, or surgical resection, and *(c)* available preoperative 3 T MRI, including APTw-MRI, DWI, and DCE-MRI. The exclusion criteria were as follows: *(a)* prior treatment history for head and neck tumors (n = 56), *(b)* no record of fine needle aspiration, biopsy, or surgery, or inconclusive pathologic results (n = 12), *(c)* final pathology not confirmed as salivary gland tumors (external auditory canal cancer, n = 1; first branchial cleft cyst, n = 1; IgG4-related disease, n = 1; intramuscular lipoma, n = 3; sebaceous adenoma, n = 1; veno-lymphatic malformation, n = 3), *(d)* lack of available APTw-MRI, DWI, or DCE-MRI (n = 42), and *(e)* inadequate MRI quality (n = 6). Demographic data were obtained via electronic medical record.

As a result, a total of 38 subjects (median age, 58 years; age range, 18–78 years; 25 females and 13 males) were finally included. Thirty-six subjects had tumors in the parotid gland, and two subjects had tumors that originated from the submandibular gland. Final histopathological diagnoses were pleomorphic adenoma (n = 16), Warthin tumor (n = 6), oncocytoma (n = 1), epithelial-myoepithelial carcinoma (n = 2), mucoepidermoid carcinoma (n = 7), salivary duct carcinoma (n = 2), secretory carcinoma (n = 1), squamous cell carcinoma (n = 2), and carcinoma ex pleomorphic adenoma (n = 1), which constituted 23 benign and 15 malignant tumors.

### Imaging protocol

MRI was performed using a 3 T instrument (Ingenia CX; Philips Healthcare, Best, the Netherlands) with a 32-channel sensitivity encoding head coil. Coronal T2-WI with fat suppression, axial T2-WI with and without fat suppression, axial T1-WI without fat suppression were obtained, followed by APTw-MRI, DWI, and DCE-MRI. The acquisition of APTw-MRI covered entire tumor portion with a reference to axial T2-WI. Lastly, axial, coronal, and sagittal post-contrast T1-WI scans with fat suppression were performed. The imaging protocols for APTw-MRI, DWI, and DCE-MRI are detailed in Supplementary Materials.

### Imaging processing of APTw-MRI

APTw-MRI processing was performed using home-developed Matlab (MathWorks, Natick, MA, USA) program. Water frequency shift was corrected based on water-saturation shift referencing images^29^. Z-spectrum for each voxel was fitted by the 12^th^ order polynomial model at all offset frequencies. Then, the fitted curve was interpolated to a higher resolution of 1 Hz. The actual water resonance frequency was assumed to be at the lowest signal of the interpolated Z-spectrum. The water center frequency offset was measured as the displacement between the actual and ideal water resonance frequency of 0 Hz. The APT Z-spectrum at six frequency offsets was interpolated over the offset range and shifted using the estimated water center frequency offset^29^. Based on the final shift-corrected Z-spectrum, asymmetric magnetization transfer ratio (MTR_asym_) analysis was performed with respect to water frequency^9^. For APTw-imaging, MTR_asym_ at 3.5 ppm was calculated as follows:

$${{\rm{MTR}}}_{{\rm{asym}}}(3.5\,{\rm{ppm}})=[{S}_{sat}(-3.5\,{\rm{ppm}})-{S}_{sat}(3.5\,{\rm{ppm}})]/{S}_{{0}},$$

where *S*_*sat*_ and *S*_0_ are the signal intensities obtained with and without selective saturation pulse, respectively. We defined APTw-signal as MTR_asym_ (3.5 ppm) × 100 (%).

### Image analysis

#### Tumor identification and ROI allocation

Two board-certified neuro-radiologists (Y.J.B. and B.S.C. with 9 and 19 years of experience, respectively), blinded to the clinical and histopathological information, independently reviewed all MRIs. First, they identified the tumors on T2-WI and post-contrast T1-WI. With reference to them, smoothed polygonal ROIs were allocated on a single section of the APTw-MRI exported to the ImageJ software (National Institute of Health, Bethesda, MD), which could best represent the entire tumor signal, covering the largest solid portion of the tumor. The readers drew ROIs, while attempting to include as much of the solid tumor as possible and exclude the cystic or necrotic portion. Lastly, a single reader (Y.J.B.) placed the ROIs on ADC map and DCE-MRI at the same section as the APTw-MRI.

#### APTw-signal measurement

APTw-signals were measured in each ROI on APTw-MRI. The following values were automatically calculated according to the histogram-based analysis: mean, minimum, maximum, and median values of APTw-signals, skewness, and kurtosis. The values of the APTw-signals from the two readers were averaged and used for further analysis.

#### DWI and DCE-MRI analysis

First, the mean ADC in each ROI was measured on the ADC map from DWI. Second, on DCE-MRI, the average signal intensities within the ROI were plotted against time to construct TIC. TICs were then classified into the following four types: type A, time to peak >120 seconds; type B, time to peak ≤ 120 seconds, high wash-out ratio (≥30%); type C, time to peak ≤ 120 seconds, low wash-out ratio (<30%); and type D, flat^3,24^. According to the previous scheme using both ADC and TIC^3^, we categorized the lesions with a >type A TIC, b >type B TIC and ADC < 1.0 × 10^−3^ mm^2^/sec, c > type C TIC and ADC ≥ 1.4 × 10^−3^ mm^2^/sec, and d > type D TIC into benign tumors; otherwise, the tumors were categorized into malignancy.

### Statistical analysis

Continuous variables were expressed as the median and range. Demographic data between benign and malignant tumors were compared using Chi-square and Mann-Whitney tests. Inter-observer agreement on APTw-signals between two readers was evaluated by ICC: greater than or equal to 0.75, excellent agreement; 0.60–0.74, good agreement; 0.40–0.59, fair agreement; and less than 0.40, poor agreement. Differences in APTw-signals, ADC values and TIC patterns between benign and malignant tumors were compared using Mann-Whitney test, Chi-square test, and linear association test. Diagnostic performances predicting malignant tumors using APTw-signals, ADC values, TIC patterns, and the results from multi-parametric analysis combining DWI and DCE-MRI, were evaluated via ROC curve analysis. The AUC values from each ROC curve analysis were compared using DeLong test^30^. *P* values of less than 0.05 were considered to indicate significant differences. All statistical analyses were performed using SPSS v. 22.0 (SPSS, Chicago, IL, USA) and MedCalc 17.9 (MedCalc, Mariakerke, Belgium).

## Supplementary information

Supplementary Materials
