# Supplementary material for: Amide Proton Transfer-weighted MRI in the Diagnosis of Major Salivary Gland Tumors
Source: Sci Rep. 2019 Jun 6;9:8349. doi: 10.1038/s41598-019-44820-0 (PMC6554276; doi:10.1038/s41598-019-44820-0)
Supplement: Supplementary file 1 — Supplementary Materials [file 41598_2019_44820_MOESM1_ESM.docx]

**Amide Proton Transfer-weighted MRI in the Diagnosis of Major Salivary Gland Tumors**

Yun Jung Bae, MD, PhD^1^, Byung Se Choi, MD, PhD^1*^, Woo-Jin Jeong, MD, PhD^2^, Young Ho Jung, MD, PhD^2^, Jung Hyun Park, MD^1^, Leonard Sunwoo, MD, PhD ^1^, Cheolkyu Jung, MD, PhD^1^, Jae Hyoung Kim, MD, PhD^1^

*^1^Department of Radiology, Seoul National University Bundang Hospital, 82, Gumi-ro 173beon-gil, Bundang-gu, Seongnam, Republic of Korea*

*^2^Department of Otolaryngology–Head & Neck Surgery, Seoul National University Bundang Hospital, 82, Gumi-ro 173beon-gil, Bundang-gu, Seongnam, Republic of Korea*

***Corresponding author**:

Byung Se Choi, MD, PhD

Department of Radiology

Seoul National University Bundang Hospital

82, Gumi-ro 173beon-gil, Bundang-gu, Seongnam, 13620, Republic of Korea

Tel: +82-031-787-7625, Fax: +82-031-787-4011

E-mail: [byungse.choi@gmail.com](mailto:byungse.choi@gmail.com)

**Competing interests**

The authors declare no competing interests

.

**Sources of Funding**

This work was supported by grant No. 02-2018-030 from the SNUBH Research Fund.

**Supplementary Materials**

**Materials and Methods: Imaging Protocol**

APTw-MRI was acquired with three-dimensional segmented echo-planar imaging (EPI) sequence readout after every short saturation RF pulse using a single-lobed Gaussian pulse with the duration of 70 msec and the peak amplitude of 2 μT. In this way, the saturation was built up over multiple saturation RF pulses as pulsed steady-state (Supplementary Fig. 4)^1^. Total 6 saturation frequency offsets (±3.0, ±3.5 and ±4.0 ppm) were adopted, and 4 repetitions were performed at ±3.5 ppm to achieve sufficient signal-to-noise ratio in a clinically reasonable time^2, 3^. Other main imaging parameters were as follows: repetition time (TR), 146 msec; echo time (TE), 7 msec; field-of-view (FOV), 210 × 210 mm^2^; acquisition voxel size, 2 × 2.5 × 6 mm^3^; EPI factor, 9; sensitivity encoding factor, 2; number of slice sections, 33; scan time, approximate 2 minutes 50 seconds. Water-frequency shift from field inhomogeneity was measured in a separate acquisition using water-saturation shift referencing (WASSR) method^4^. This direct water saturation imaging was utilized in the measurement of the absolute water frequency in each voxel, allowing appropriate centering of Z-spectrum on a voxel-by-voxel basis, independent of spatial B0 field inhomogeneity^4^. The off-resonance saturation frequency for WASSR image was performed throughout a range between -1.5 and 1.5 ppm with intervals of 0.5 ppm. Other parameters for the WASSR image were as follows: TR, 146 msec; TE, 7 msec; FOV, 210 × 210 mm^2^; acquisition voxel size, 2 × 2.5 × 6 mm^3^; flip angle, 25°; EPI factor, 9; sensitivity encoding factor, 2; number of slice sections, 33; scan time, approximate 4 minutes 5 seconds.

Single-shot EPI-based DWI was performed in the axial plane using 3 orthogonal diffusion gradients with b-values of 0 and 1000 s/mm^2^. The following parameters were adopted: TR, 6700 msec; TE, 75 msec; FOV, 220 × 220 mm^2^; acquisition matrix, 128 × 128; slice thickness, 3 mm; number of slice sections, 40; scan time, approximate 2 minutes 20 seconds. ADC map was generated accordingly.

DCE-MRI was based on three-dimensional fast field echo sequence. After intravenous injection of 0.1 mmol/kg of gadobutrol (Gadovist®, Bayer Healthcare, Berlin, Germany), 65 dynamic scans of axial T1-WI were obtained at an interval of 6 seconds using the following parameters: TR, 6 msec; TE, 3 msec; FOV, 180 × 240 mm^2^; acquisition matrix, 192 × 192; slice thickness, 6 mm; slice overlap, 3 mm; flip angle, 8°; number of slice sections, 24; scan time, approximate 6 minutes 12 seconds.

**References**

1. Jones, C.K. et al. In vivo three-dimensional whole-brain pulsed steady-state chemical exchange saturation transfer at 7 T. *Magn Reson Med* 67, 1579-1589 (2012).

2. Joo, B. et al. Amide proton transfer imaging for differentiation of benign and atypical meningiomas. *Eur Radiol* 28, 331-339 (2018).

3. Zhou, J. et al. Three-dimensional amide proton transfer MR imaging of gliomas: Initial experience and comparison with gadolinium enhancement. *J Magn Reson Imaging* 38, 1119-1128 (2013).

4. Kim, M., Gillen, J., Landman, B.A., Zhou, J. & van Zijl, P.C. Water saturation shift referencing (WASSR) for chemical exchange saturation transfer (CEST) experiments. *Magn Reson Med* 61, 1441-1450 (2009).

**Supplementary Figures and Figure Legends**

**
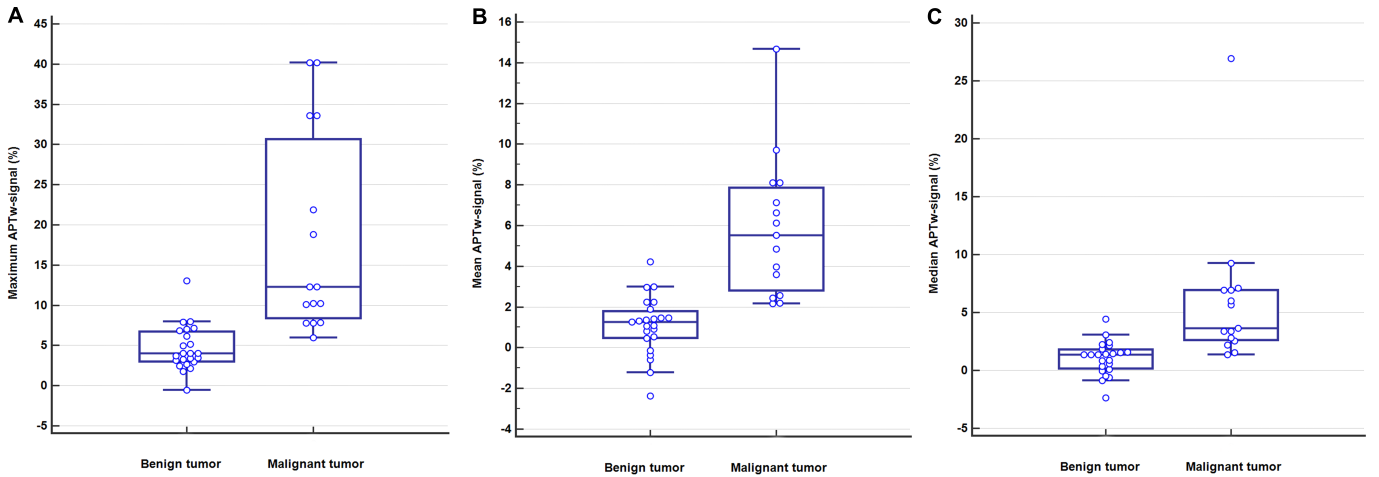
**

**Supplementary Figure 1. Box and whisker plots for (A) maximum, (B) mean, and (C) median amide proton transfer-weighted (APTw)-signals in benign and malignant tumors of the major salivary glands.**

The bottom and top of the box are the first and third [quartiles](http://en.wikipedia.org/wiki/Quartile); the band inside the box represents the [median](http://en.wikipedia.org/wiki/Median) value; and the thin solid line through the box represents the difference between the maximal and minimal values. All data are plotted with circles. Graphs show that the maximum, mean, and median APTw-signal values are significantly higher in malignant tumors than in benign tumors. Detailed data are present in the “Results” section.

**
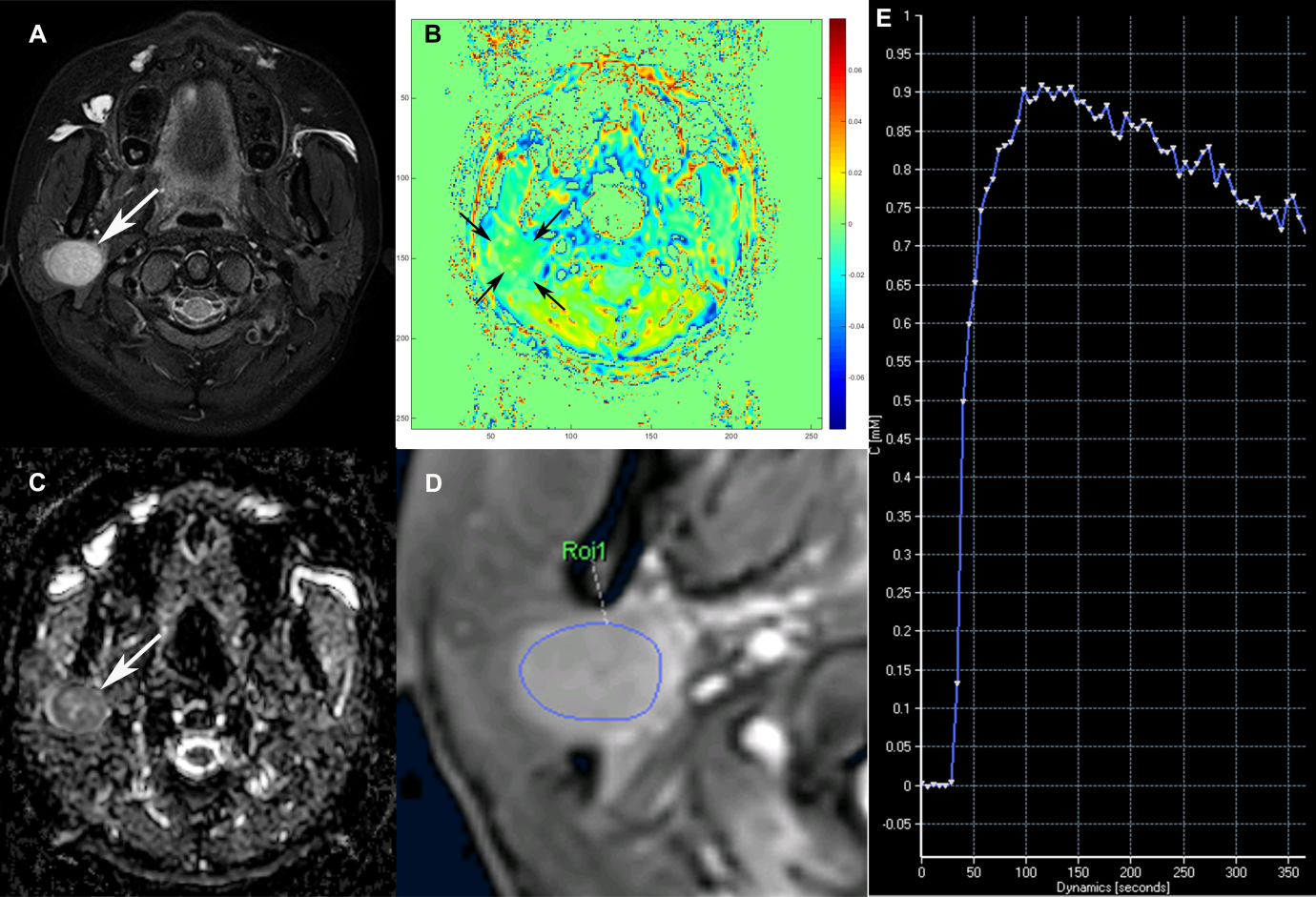
**

**Supplementary Figure 2. Pleomorphic adenoma of a 25-year-old female.**

**(A)** Axial T2-weighted imaging with fat suppression shows a well-defined solid mass with T2-hyperintense signal intensity in the right parotid gland (arrow).

**(B)** Amide proton transfer-weighted (APTw)-MRI demonstrates that APTw-signal of the tumor is relatively low (arrows). The averaged APTw-signal values from the two readers in the solid portion were: Mean APTw-signal, 1.46%; maximum APTw-signal, 4.02%; median APTw-signal, 1.5%.

**(C)** The apparent diffusion coefficient (ADC) map shows relatively low ADC value in the solid portion of the tumor (arrow). Mean ADC value in the solid portion of the tumor was 1.28 × 10^-3^ mm^2^/sec.

**(D)** The region-of-interest (ROI) is located in the solid portion of the tumor on dynamic contrast-enhanced (DCE)-MRI for the generation of time-intensity curve (TIC).

**(E)** Resultant TIC represents type C with time-to-peak of less than 120 seconds and low washout ratio (< 30%). Therefore, multi-parametric analysis, based on ADC and TIC, led to the false-positive diagnosis of the lesion as malignant.


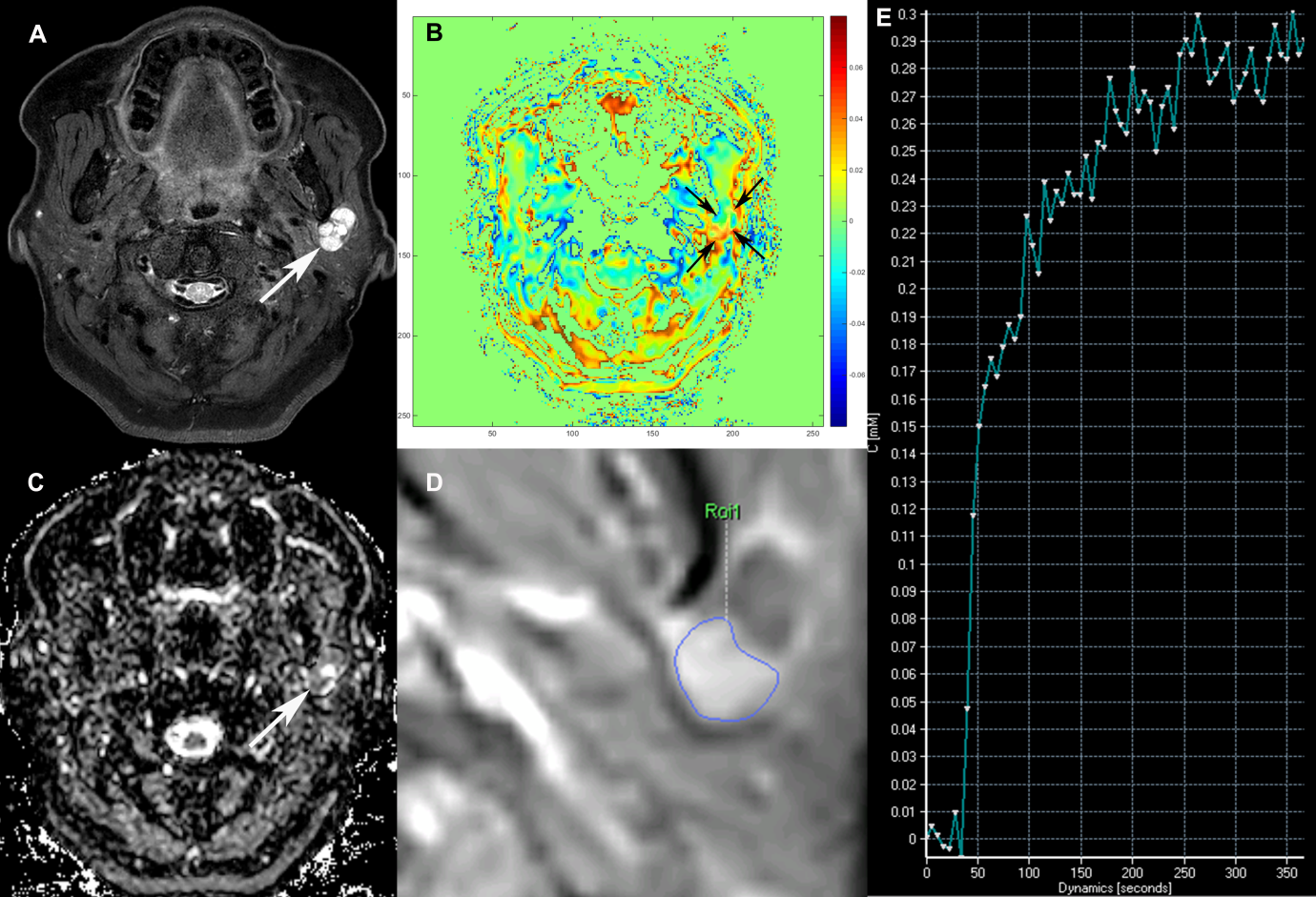


**Supplementary Figure 3. Epithelial-myoepithelial carcinoma of a 71-year-old male.**

**(A)** Axial T2-weighted imaging with fat suppression shows a well-defined hyperintense mass with focal cystic portion in the left parotid gland (arrow).

**(B)** Tumor shows heterogeneous signal on amide proton transfer-weighted (APTw)-MRI (arrows). Note focal increase in the asymmetry value at the solid portion of the tumor. The averaged APTw-signal values obtained by the two readers in the solid portion were: Mean APTw-signal, 2.15%; maximum APTw-signal, 9.66%; median APTw-signal, 1.53%.

**(C)** The apparent diffusion coefficient (ADC) map shows relatively low ADC value in the solid portion of the tumor (arrow). Mean ADC value in the solid portion of the tumor was 1.31 × 10^-3^ mm^2^/sec.

**(D)** The region-of-interest (ROI) is located in the solid portion of the tumor on dynamic contrast-enhanced (DCE)-MRI for the generation of time-intensity curve (TIC).

**(E)** Resultant TIC represents persistent enhancement pattern with time-to-peak of more than 120 seconds (type A). Therefore, multi-parametric analysis, based on ADC and TIC, led to the false-negative diagnosis of the lesion as benign.

**
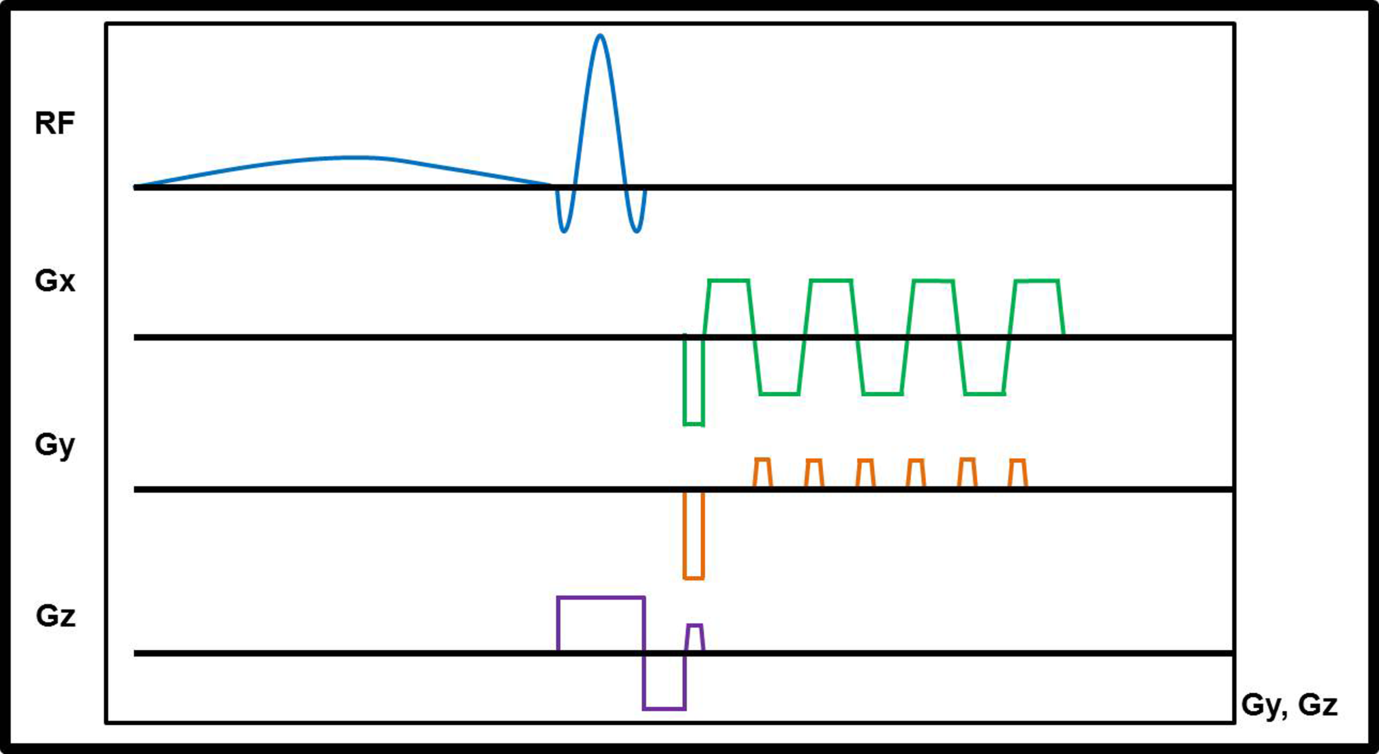
**

**Supplementary Figure 4. Pulse sequence for amide proton transfer-weighted MRI (APTw)-MRI.**

Pulsed chemical exchange saturation transfer acquisition consists of a frequency-selective sinc-gauss saturation pulse, followed by a short partial echo-planar imaging readout. This repetition time (TR) interval is repeated continuously with the saturation pulse applied at one frequency to fill 3D k-space during the stead state building up. This sequence diagram was modified from Jones, C.K. et al.^1^.
